# Contrast transfer function correction applied to cryo-electron tomography and sub-tomogram averaging

**DOI:** 10.1016/j.jsb.2009.08.002

**Published:** 2009-11

**Authors:** Giulia Zanetti, James D. Riches, Stephen D. Fuller, John A.G. Briggs

**Affiliations:** a Structural and Computational Biology Unit, European Molecular Biology Laboratory, Meyerhofstrasse 1, Heidelberg, Germany; b University of Oxford, Division of Structural Biology, Wellcome Trust Centre for Human Genetics, Henry Wellcome Building for Genomic Medicine, Oxford, United Kingdom

**Keywords:** Cryo-electron tomography, Sub-tomogram averaging, CTF correction, PRD1

## Abstract

Cryo-electron tomography together with averaging of sub-tomograms containing identical particles can reveal the structure of proteins or protein complexes in their native environment. The resolution of this technique is limited by the contrast transfer function (CTF) of the microscope. The CTF is not routinely corrected in cryo-electron tomography because of difficulties including CTF detection, due to the low signal to noise ratio, and CTF correction, since images are characterised by a spatially variant CTF. Here we simulate the effects of the CTF on the resolution of the final reconstruction, before and after CTF correction, and consider the effect of errors and approximations in defocus determination. We show that errors in defocus determination are well tolerated when correcting a series of tomograms collected at a range of defocus values. We apply methods for determining the CTF parameters in low signal to noise images of tilted specimens, for monitoring defocus changes using observed magnification changes, and for correcting the CTF prior to reconstruction. Using bacteriophage PRD1 as a test sample, we demonstrate that this approach gives an improvement in the structure obtained by sub-tomogram averaging from cryo-electron tomograms.

## 1 Introduction

The three-dimensional (3D) structure of biological specimens such as proteins, protein complexes or viruses can be determined to high resolution by cryo-transmission electron microscopy. TEM images can be approximated to 2D projections of the specimen; its 3D structure can be obtained by combining projection views from different angles. In single particle experiments, multiple individual images of untilted specimens are sufficient for obtaining a reconstruction (see Zhou (2008) for a recent review). In cryo-electron tomography (CET) the projection views are obtained by tilting the specimen holder and collecting a series of images of the same object at defined angular increments (a “tilt series”). A 3D reconstruction is then calculated, normally using weighted back-projection. CET presents advantages when dealing with heterogeneous samples, because the 3D reconstruction can be obtained from a single object (Lucic et al., 2005). Vitrified biological specimens are sensitive to beam damage, and the tolerable electron dose must hence be distributed over the tilt series. In each image of a CET tilt series the electron dose is usually of the order of 1–2 e^−^/Å^2^, as a consequence, the signal to noise ratio (SNR) is particularly low.

The resolution of tomographic reconstructions of vitrified biological specimens is anisotropic because the maximum tilt-angle is limited by the slab geometry of the sample holder. The isotropic, high SNR structure of a protein or protein complex can be obtained by averaging multiple sub-tomograms containing identical copies of the complex studied (Briggs et al., 2009; Forster and Hegerl, 2007; Forster et al., 2005; Liu et al., 2008; Zanetti et al., 2006; Zhu et al., 2006). CET combined with sub-tomogram averaging is hence the technique of choice when aiming at elucidating the 3D structure of protein complexes *in situ*, where their 2D projections overlap with cellular or viral elements and the complexes can be computationally extracted and analysed only upon 3D reconstruction.

The individual images in the tomographic tilt-series are modulated in a spatial frequency-dependent manner by the contrast transfer function (CTF), described by Eq. (1.0) (Erickson and Klug, 1971; Wade, 1992)

(1.0) $$\text{CTF}(f)=A(\sin(\pi \lambda f^{2}(\Delta z-0.5\lambda^{2}f^{2}c_{\text{s}}))+B\cos(\pi \lambda f^{2}(\Delta z-0.5\lambda^{2}f^{2}c_{\text{s}})))$$

where the independent variable is the spatial frequency (*f*), Δ*z* is the defocus, *c*_s_ is the spherical aberration, *λ* is the electron wavelength. *A* is a defocus-dependent envelope function describing the decay of the signal for an instrument with a given angular aperture (Wade, 1978, and Supplementary materials), and *B* is the fraction of amplitude contrast.

The microscope parameters do not usually vary within an imaging session, except the defocus. In order to obtain a faithful representation of the 3D object it is necessary to correct the signal, by phase flipping or Wiener filtering (Frank, 2006). Since the oscillatory nature of the CTF causes the signal intensity to be zero at certain spatial frequencies, different defocus values must be combined for complete restoration. In single particle reconstruction several images must be collected at different defoci, whereas this is not strictly necessary for images of tilted specimens which are characterised by a defocus gradient (Winkler and Taylor, 2003). In order to perform CTF correction appropriately it is necessary to determine the shape of the CTF for each image contributing to the reconstruction. Typically, an approximate shape for the function is calculated using expected values for the parameters. The parameters are then refined by fitting a theoretical CTF curve into the rotationally averaged power spectrum of the image. This approach becomes unfeasible when the SNR in the power spectrum is too low to detect the CTF oscillations. This is the case in CET where the electron dose must be distributed over several images.

To achieve sufficient contrast for image processing purposes in CET, the defocus values used are typically higher than those used for single particle reconstruction. At a defocus of 4 μm on a 300-kV microscope, the CTF has its first node at about 1/28 Å^−1^. The resolution of the reconstruction will be limited to this spatial frequency if the CTF is not corrected. At this intermediate resolution tentative fitting of atomic structures into the electron density map can be incorrect, and it is desirable to perform CTF correction in order to increase the resolution of the reconstruction.

Substantial difficulties are encountered in the process of CTF-correcting cryo-tomographic data: first, instrumental inaccuracies do not guarantee that the defocus of each image in a tilt series is maintained. Second, the low SNR hinders detection of the CTF signal on individual images. Third, images of tilted specimens present a defocus gradient, and CTF correction cannot be applied uniformly to the whole image.

Here we demonstrate the feasibility of applying CTF correction to obtain higher resolution reconstructions using sub-tomogram averaging. For clarity, we consider the problem in four stages:

1. Simulations of the effects of the CTF, and of CTF correction.

The assumption that the defocus is the same for all images might not be correct. After measuring the defocus on tilt series of a carbon film sample we find that the holder instability can cause shifts in the defocus at the tilt axis of up to 2.5 μm. Here we simulate the effect of inaccuracies in defocus determination when correcting tilt images. We demonstrate that an imprecise defocus determination can still allow CTF correction to give a substantial improvement in resolution.

2. Approaches for measuring and approximating the defoci of the images.

The low SNR on cryo-tomographic images does not permit the identification of the signal oscillations attributable to the CTF directly from the power spectrum. Several methods for detecting the CTF in tilted specimens have been proposed in the past. Mindell and Grigorieff (2003) calculate the power spectrum of sub-regions extracted from the image to give an estimate of local defocus. This approach is based on data collected with the electron dose used for single particle studies, usually about 20 e^−^/Å^2^, which generates images with sufficient SNR to detect CTF oscillations. Winkler and Taylor (2003) rotate the images with their tilt axis horizontal, so that image strips with almost invariant defocus can be used to compute the rotationally averaged power spectrum. The latter study is performed on plastic embedded samples, which are again characterised by a SNR higher than cryo-samples. Fernandez and Crowther (Fernandez et al., 2006) determine the CTF of tilted cryo-images by strip based periodogram averaging. The defocus at the tilt axis is assumed to be the same for all images in a tilt series. Areas of similar defocus value are extracted from all images and their mean power spectrum calculated. The defocus values used in the latter study ranged between 6 and 26 μm, which are particularly high, even for CET. None of the published methods have been applied to CET and sub-tomogram averaging for defocus values lower than 5 μm.

3. Approaches for correcting the CTF.

Another challenge in the process of CTF correction in tomographic images is posed by the defocus gradient perpendicular to the tilt axis, as correction of the whole image with a uniform CTF is not appropriate. Several studies have proposed methods to correct the CTF on images of tilted specimens. For crystalline specimens, Henderson et al. (1990) have expressed the CTF at each spatial frequency as a function of the defocus (the tilt transfer function), and they used it to correct areas of the Fourier transform of the crystal images. This approach is not well suited for non-crystalline specimens, where the information is distributed continuously on all spatial frequencies. Fernandez et al. and Winkler et al. (Fernandez et al., 2006; Winkler and Taylor, 2003) both propose a strip based correction, in which the image is rotated with the tilt axis horizontal, and correction of strips is performed after interpolation. In this paper, we divide the tilted image into tiles, and perform CTF correction prior to rotation.

4. Testing of these approaches in sub-tomogram averaging of cryo-tomograms of bacteriophage PRD1.

We test our method on averaged sub-tomograms of the bacteriophage PRD1 (Abrescia et al., 2004). Our results show an increase in resolution upon CTF correction from 29 to 22 Å, as judged from the Fourier shell correlation between half datasets at the 3*σ* threshold criterion. We also see a significant improvement in the correlation between the averaged tomographic map and the electron density derived from the atomic structure of the PRD1 capsid (Abrescia et al., 2004). This is reflected in improvement of the quality of the electron density maps.

## 2 Materials and methods

The PRD1 bacteriophage samples were grown in DS88 (*Salmonella enteritica serovar typhimurium*) (Bamford and Bamford, 1990), and purified via sucrose gradient centrifugation before pelleting and resuspension in potassium phosphate buffer (pH 7.2) (Bamford and Bamford, 1991; Walin et al., 1994). The sample was mixed with protein-A colloidal gold, suspended in PBS and plunge frozen with the Vitrobot (FEI, Eindhoven, The Netherlands). Three microliters were deposited on a glow discharged C flat grid (2 μm holes with 1 μm spacing on 300 mesh, Protochips, Raleigh, North Carolina, United States). Carbon film samples were prepared by depositing diluted gold solution deposited on a continuous carbon film.

Data were recorded using an FEI Tecnai F30 ‘Polara’ transmission electron microscope (FEI, Eindhoven, The Netherlands), equipped with a 300-kV field emission gun, Gatan GIF 2002 post-column energy filter, and 2k∗2k Multiscan CCD camera (Gatan, Pleasanton, California, United States). All data collection was performed at 300 kV, with the energy filter operated in the zero-loss mode. Tilt series were typically recorded from −63° to +63° with an angular increment of 3°. The total dose was between 45 and 70 e^−^/Å^2^, higher tilt images were imaged with longer exposure times in order to account for the increased thickness. The defocus was between 3 and 5 μm. The nominal magnification was 27,500 times, which corresponded to a pixel size of 4.7 Å at the specimen level.

Tilted images were aligned using gold beads as fiducial markers to within a maximum bead positioning error of less than 1 pixel (4.7 Å). The bead positioning error was marginally higher for beads further from the centre of the tomogram, with a mean error of 0.64 pixel for the closest half of the beads and a mean error of 0.74 for the furthest. Three-dimensional reconstructions were obtained using weighted back projections. Alignment of the tilt series and tomographic reconstruction were performed using the IMOD software package (Kremer et al., 1996).

All simulations and sub-tomogram processing was performed using MATLAB (Mathworks, Natick, Massachusetts, USA). Scripts for sub-tomogram alignment and averaging were adapted from the TOM and AV3 software packages (Forster et al., 2005; Nickell et al., 2005). Scripts for CTF correction are described in the results section. On a modern linux workstation correction of a whole tomogram requires 4–5 min when sampling every 128^2^ pixels and calculating 256^2^ FFTs, and more than 10 times as much if the images are sampled every 32^2^ pixels. The resolution was assessed by Fourier shell correlation (FSC). Datasets were divided in halves and the FSC between the maps obtained by averaging each half was calculated using Imagic (van Heel et al., 1996). A simple spherical-shell mask with inner and outer radii of 45 and 80 pixels (211.5 and 376 Å) and an edge softened over 20 pixels (94 Å) was applied to both maps prior to computing the FSC.

FSCs were also calculated between the averaged tomographic maps and the electron density map obtained from the crystal structure. An electron density map of the PRD1 capsid shell was created from the atomic model of PRD1 asymmetric unit (PDB 1W8X) containing the P3, P30, P31, and P16 proteins only. To do this, adapted model_fc.inp and model_map.inp input files from the program CNS were used, and icosahedral symmetry was applied to recreate electron density correspondent to a whole virion. The electron density was scaled to the same pixel size as the tomographic maps for comparison, and prior to computing the FSCs, the atomic model-derived map was aligned to the average of corrected and uncorrected tomographic maps, after filtering the EM data to 30 Å. The maps were masked with the soft-edged spherical shell described above.

## 3 Results

### 3.1 Simulations of the effects of the CTF, and of CTF correction

#### 3.1.1 Stability of defocus in a tomographic series

**Fig. 1:**
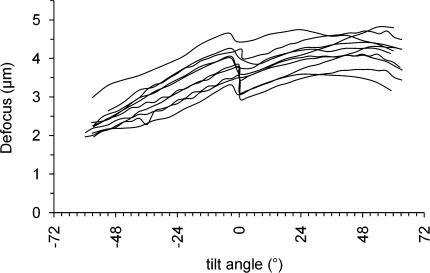
Defocus change over tilt series. Defocus measured from images of 10 tilt series of a carbon film, shown as a function of the tilt angle.

In an electron microscope, the amount of defocus, for a set of lenses with invariant current, depends on the position of the object along the optical axis (the *z* axis in the convention used here), and can be changed by moving the specimen holder along this axis. The mean defocus characterising an image of a tilted specimen corresponds to the defocus at the image’s central point. During the acquisition of a tilt series the holder is rotated about its eucentric axis. If the physical tilt axis of the specimen does not intersect the centre of the image, or if the *z*-position of the physical tilt axis does not remain constant throughout a tilt series, the mean defocus value of an image changes at different tilt angles. To assess the extent of the defocus variation in the data considered in this paper, we collected a number of high-dose tilt series of a carbon film sample, and measured the defocus of each of the images. The software package we have used for data collection, UCSF Tomography (Zheng et al., 2007), collects the first half of the tilt series starting from the flat 0° position by increasing the tilt angle in one direction, and then rotates the holder back to zero degrees to collect the second half of the tilt series, in the opposite direction. The software does not measure the defocus after each tilt image. The mean defocus changed within a tilt series, as shown in Fig. 1. Most series show a jump in defocus between the two halves of the series. The range of defocus within a tilt series was between 1.5 and 2.5 μm. The pattern of change is similar in all series collected with the same software, but the exact value of defocus is not predictable.

#### 3.1.2 Simulation of the effect of errors in defocus determination on CTF correction

Defocus variations within a tilt series originate from both changes in the *z*-position of the sample at the centre of the image and from the tilting of the specimen. The tilt produces a defocus gradient across the image which can be calculated by knowing the tilt axis and angle. The shifts in the *z*-height throughout a tilt series cannot be determined experimentally because the SNR of individual images is too low to measure the defocus. To determine how the variations in defocus due to *z*-shifts may affect our ability to perform CTF correction we have simulated the effects of errors in defocus determination on a tomographic reconstruction.

Simulations were performed using defocus, tilt angles and number of tilt images from some of the datasets in Fig. 1. We assumed that a final reconstruction, after sub-tomogram averaging, is formed from particles placed in random areas of the tomogram, in random orientations. To simulate the signal modulation by the CTF applied onto the final reconstruction, we calculated the mean CTF from all sub-regions of all images in one or more tilt series. This mean CTF approximates the 3D CTF characterising the averaged structure.

In order to do this, we applied the following steps:

(a) We divided each 2048^2^ image in a tilt series into 128^2^ tiles.
(b) We calculated a defocus value for each tile based on its distance from the tilt axis, the tilt angle and the mean defocus of the image. The tilt angles and mean defocus values for each of the images in the series were from datasets in Fig. 1.
(c) Using the calculated defocus values, CTF functions were computed for all tiles in all images in the tilt series, and the mean CTF was calculated. This mean function simulates the CTF modulating the final reconstruction after sub-tomogram averaging in the absence of correction.
(d) For each tile, a defocus value for correction was determined. In some simulations this corresponded with the true defocus value used for generating the CTF (appropriate correction). Other simulations were carried out in which the defocus value for correction was different from the value calculated in step c because the tilt of the images was not taken into account, or because the *z*-shifts due to holder instabilities were not considered, or because a random error in the determination of defocus at the tilt axis was simulated.
(e) The CTF functions computed in step c for each tile were phase flipped within frequency ranges calculated based on the defocus values determined in step d. The flipped CTFs from all tiles of all images of all tomograms were then averaged. This mean function simulates the effect of the CTF on the final reconstruction after sub-tomogram averaging when appropriate or approximate CTF correction has been applied.

When averaging sub-tomograms distributed throughout the tomogram with random orientations, the mean CTF approximates the 3D CTF characterising the averaged structure.

**Fig. 2:**
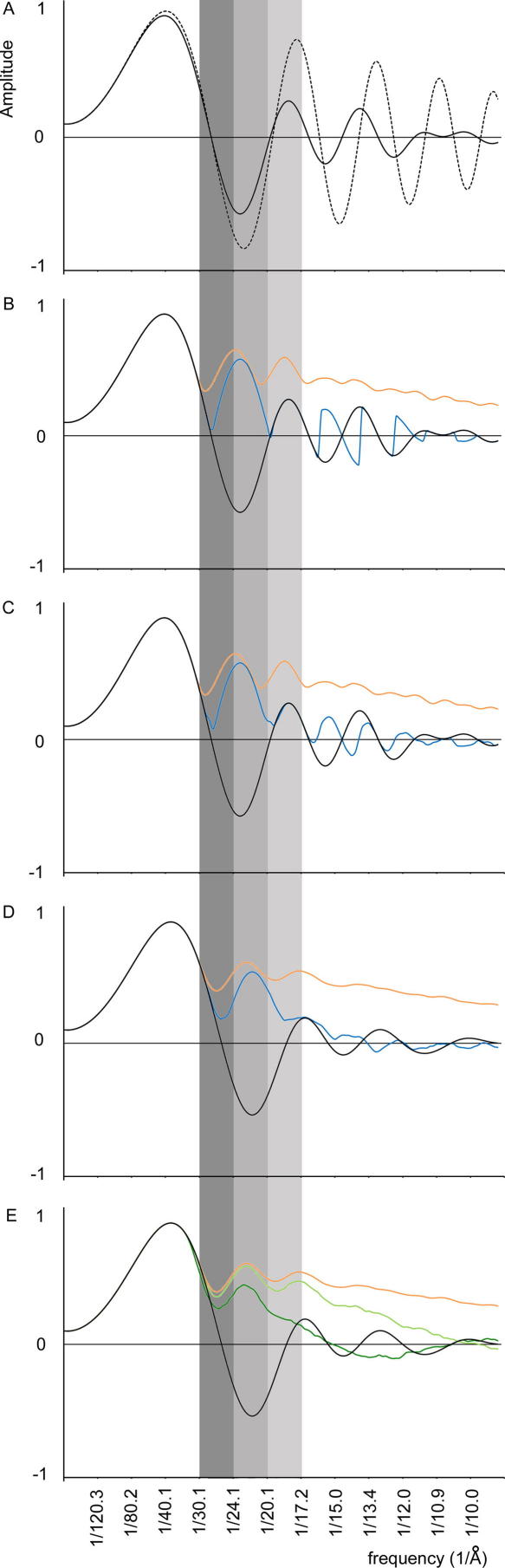
Simulation of the CTF effects on a tomographic reconstruction. (A) The CTF of a reconstruction from a single uncorrected tomogram (solid line), and the CTF of a single untilted image with a defocus equal to the mean defocus of the tomogram (dotted line). (B) Black solid line as in (A). The CTF of a reconstruction from a single tomogram corrected accurately (orange line), or corrected based on the mean defocus of the series (blue line), without taking the tilt into account. (C) Black solid line as in (A). The CTF of a reconstruction from a single tomogram corrected accurately (orange line), or corrected based on the mean defocus of the series with the tilt taken into account (blue line). (D) The same curves as in (C) for a reconstruction calculated using data from five tomograms collected at a range of defoci. (E) The CTF of a reconstruction from five tomograms, corrected based on a defocus measured with an error of ±0.5 μm (light green line) or ±1.2 μm (dark green line). Black and orange lines as in (D). See text for further details. Three resolution ranges are highlighted with vertical grey bars for clarity.

We first calculated the effect of the uncorrected CTF on a tomographic reconstruction. In Fig. 2A, the simulated CTF for a reconstruction from a series with mean defocus value of 3.94 μm and a variation of defocus at the tilt axis due to holder instabilities of 2.43 μm is shown (solid curve). The dotted curve represents a theoretical CTF curve according to Eq. (1.0), with the following parameters: voltage = 300 kV, spherical aberration = 2 mm, fraction of amplitude contrast = 10% (see Supplementary materials), defocus = 3.94 μm (the mean defocus of the series). The two functions oscillate approximately in phase up to a spatial frequency of 1/20 Å^−1^. The first node of the two functions occurs at the same spatial frequency of 1/28 Å^−1^. The limit to the resolution achievable from an uncorrected set of images collected at a range of different defoci is hence determined by the position of the first zero of the CTF at the mean defocus value. Whereas the first node of the CTF calculated at a single defocus value reflects absence of information, the corresponding node in the function describing the mean of all CTFs results from summing positive with negative contributions, and can be recovered by CTF correction of the contributing images.

The effect of CTF correction was simulated on the same dataset. Each tile containing the previously defined CTF was corrected by phase flipping before calculating the mean CTF. In a first experiment all tiles were corrected using their exact defocus values: the information in the 3D reconstruction was transmitted with the same sign in the entire frequency domain (Fig. 2B, orange line).

We next simulated the effect of errors in the estimation of the defocus for each image. The simplest correction approach is to correct every tile using the mean defocus value of the entire tilt series, thereby assuming there is no *z*-shift (i.e. variation of defocus at the tilt axis) during data acquisition, and ignoring the tilting of the images (Fig. 2B, blue line). For the tilt series used here, this gives in a maximum error in the defocus estimation of 1.2 μm. After correction, the information between the first and second nodes now has the correct sign. The nodes themselves are not eliminated by flipping the curve at an invariable frequency. At higher frequencies, small regions of inverted signal are seen between the nodes of the uncorrected mean CTF (solid line in Fig. 2A) and of the CTF used for correction (dotted line in Fig. 2A).

A better approach is to assume that the mean defocus of each image corresponds to the mean defocus of the series, but to take the tilt geometry into account when calculating the defocus of each tile. This approach (Fig. 2C, blue line) results in a partial gain of signal at the nodes. The frequency at which the phase flip is performed is slightly different for each tile, and if the direction of the tilt is set correctly, the positive contributions outweigh the negative contributions. Nevertheless, the defocus range covered within an image of a tilted specimen is quite low, and the information close to the CTF nodes remains weak.

A recovery of the signal at all frequencies is expected when a number of tomograms with significantly different defocus values are combined. In Fig. 2D, the effect of combining data from five tilt series (from Fig. 1) with defocus values ranging between 3 and 4 μm is shown. An analogous simulation was performed for tomograms in which the defocus values ranged between 2 and 3 μm, and is reported in Supplementary Fig. S2. Accurate correction is shown as an orange curve, and correction based on the mean defocus value, and considering the tilt geometry, is shown as a blue curve. Signal is transferred with the correct sign up to a spatial frequency of about 1/14 Å^−1^. This indicates that an increase in resolution can be obtained by using the mean defocus value of a tilt series to correct all of the images, even with the large variation in *z*-height characteristic of our system. If the variation in the *z*-height during data acquisition is reduced, by using a more stable holder for example, the error associated with attributing the same mean defocus value to all images in a tilt series is smaller, and higher resolutions can be achieved. This is illustrated in Supplementary Fig. S3.

A different approach we tested relies on the ability of estimating the defocus value at the tilt axis of each of the images in the tilt series with a certain approximation. Fig. 2E shows the effect of a random error in the defocus determination for each image of 0.5 μm (light green line), and 1.2 μm (dark green line) when five tomograms are combined. When compared to properly corrected dataset the efficiency of contrast transfer becomes progressively lower at increasing spatial frequencies. From our simulations it is not possible to predict at which resolution the signal to noise ratio allows the Fourier shell correlation between two half datasets to be significant, because this depends on several factors such as the number of particles averaged. An error in the determination of the defocus of 1.2 μm allows a potential improvement in resolution with respect to the absence of any CTF correction similar to that obtained for the correction of all images using the mean defocus value of the series. The result of the simulation performed with an error in the defocus determination of 0.5 μm shows that up to spatial frequencies of about 1/18 Å^−1^ the efficiency of contrast transfer is comparable with the properly corrected data. The simulations described above suggest that it is possible to improve the resolution of a structure derived from sub-tomogram averaging using CTF correction, even if only the mean defocus value of the series can be determined, or the defocus value of each image can be approximately estimated.

### 3.2 Approaches for measuring and approximating the defoci of the images

#### 3.2.1 Measuring mean defocus of a tilt series

**Fig. 3:**
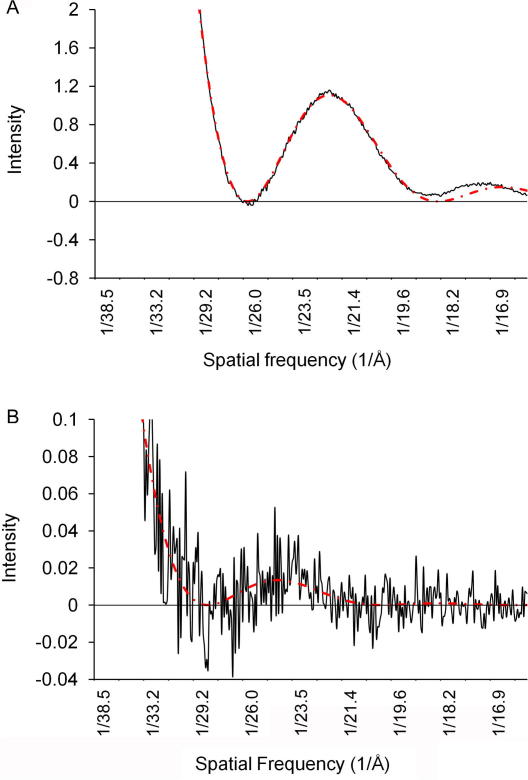
CTF fitted in the mean power spectrum of tilt series. The mean of rotationally averaged, background subtracted power spectra of all images of a tilt series were calculated (black lines). A CTF curve (dotted red line) was fitted using least square deviation methods. (A) Example from a tilt series of carbon film. The mean defocus value of the series is 3.5 μm. (B) Example from a cryo-tilt series of PRD1. The mean defocus value is 4 μm. The *y* axis in (B) is scaled up 20 times with respect to (A).

As shown in Fig. 2A, for tilt series which reflect the conditions of real data collection, the curve which describes the mean of the CTFs in a tilt series approximately overlaps at low frequencies with the CTF of a single image at the mean defocus value. This suggests that the standard method for defocus determination in cryo-EM: fitting the curve described by Eq. (1.0) to the mean of the rotationally averaged power spectra of the tiles, could be applied to the tomographic series if sufficient signal was present, and if only the low frequency region of the curve was considered. We tested this on a number of tilt series of a carbon film. The mean defocus value for a tilt series was determined by fitting a standard CTF curve to the mean of the power spectra using a least square curve fitting algorithm (Fernando, personal communication). One example is shown in Fig. 3A, where a CTF curve is fitted to the experimental data obtained by calculating the mean of the power spectra of images in a tilt series and subtracting the background. The defocus value is 3.4 μm. The mean defocus value of individual images from the same tilt series measured using the EMAN program CTFIT (Ludtke et al., 1999) is 3.3 μm. Considering 10 tilt series the error in the defocus estimate was 0.3 ± 0.2 μm.

The same method was then applied to a number of cryo-tilt series of a test sample, the bacteriophage PRD1 (an example is shown in Fig. 3B). Despite the very low SNR, one significant oscillation can be fitted by the CTF curve. For our data, we were unable to obtain a measurement of the defocus using the TOMOPS and TOMOCTFFIND programs (Fernandez et al., 2006).

#### 3.2.2 Measuring defocus on individual images using their relative magnification

We next attempted to improve our estimate of the mean defocus of individual images based on their relative magnifications. In a system with non-parallel illumination, defocus and magnification are linearly related for unvarying Condenser 2 lens settings (see Supplementary materials; van Duinen et al., 2005). The gradient depends mainly on the convergence of the beam on the specimen, i.e. the strength of the Condenser 2 lens. For an over-focused condenser lens, specimens at higher defocus have lower magnification.

**Fig. 4:**
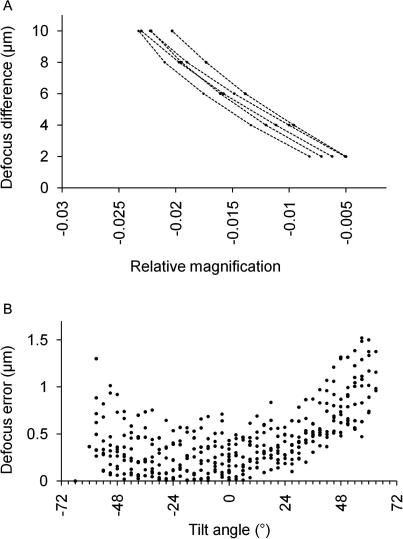
Relationship between magnification and defocus. (A) The change in defocus is plotted against the relative magnification for a series of images of carbon collected at settings of C2 and beam intensity corresponding to those used in cryo-tomography experiments. Changes in defocus and magnification are relative to the closest to focus image. (B) Error in defocus determination from nine series of carbon film. Defocus values were measured directly from the images and calculated using their relative magnification values, and the two estimates subtracted. The absolute value of the error is plotted against tilt angle.

To determine the defocus/magnification ratio at our cryo-tomographic experimental settings, series of images of gold beads on carbon film were collected. Within each series of images, the defocus was varied by changing the *z*-height of the microscope stage, thereby moving the sample relative to the objective lens. By measuring defocus and magnification changes throughout these series the defocus/magnification ratio for various Condenser 2 lens conditions could be assessed. The magnification was measured by comparing the positions of about 10 gold beads in all images. The results are shown in Fig. 4A. The data are well fitted by linear equations (reported in the Supplementary materials). The gradient of the line is reproducible within each dataset and the absolute value of the gradient is higher for lower dose conditions, consistent with what is predicted by the geometry of the system (Supplementary materials). At the microscope settings used for CET we obtained a value for the gradient of −440 μm.

This was then tested on tilt series of carbon film collected at high dose, for which the mean defocus value was measurable at each tilt by fitting a CTF curve to the oscillating signal. The magnification was measured using IMOD, where it can be iteratively refined together with the tilt angle and the direction of the tilt axis until the alignment error between subsequent images is minimised. A defocus value was obtained for each image from its magnification using the defocus measured from the mean power spectrum as a reference. The calculated values were then compared with the defocus measured directly from each image. The average error in the determination of the defocus value is less than 0.5 μm, although it increases at higher tilt angles (Fig. 4B). An error in defocus determination might result if the magnification is measured on gold beads which have a centre of gravity that does not correspond to the centre of the tomogram, and this effect is maximised at high tilt angles. It is hence important that gold beads are well distributed on the tomogram. Knowing the magnification change for all images and the mean defocus value of the series, it is therefore possible to estimate the defocus at the tilt axis of all the images in a tilt series.

### 3.3 Approaches for correcting the CTF

We developed a series of scripts for correcting images within a tilt series with a defocus gradient in a tile-by-tile based approach. These apply CTF correction to tilted images prior to image rotation. For each tilt series, a parameter file adapted from the IMOD alignment log file is generated, in which the direction of the tilt axis, the tilt angle, and the relative magnifications are defined. The mean defocus value of the series is also provided. The scripts assign the defocus value at the tilt axis of each image either by using the mean defocus value of the tilt series, or by calculating it from the relative magnifications.

The image is then divided into a number of tiles of a specified size, for example 128^2^ pixels. For each tile, the defocus value is calculated based on the position of the central pixel and on the defocus at the tilt axis. A square box is then extracted from the image around each tile. The box must be bigger than the tile, for example 256^2^ pixels. The Fourier Transform of the box is calculated and corrected for the appropriate CTF by phase flipping. The central 128^2^ pixels of the corrected box are then pasted into the corrected final image. The errors due to approximation of the tile to a flat sub-image can be reduced by choosing a smaller tile size, although increasing computational time can be a limiting factor.

### 3.4 Testing of these approaches in sub-tomogram averaging of cryo-tomograms of bacteriophage PRD1

#### 3.4.1 Sub-tomogram averaging of PRD1

The CTF detection and correction methods described above were applied to a set of cryo-tomograms of PRD1. This bacteriophage was used as a test sample since its structure is known from previous crystallography and electron microscopy studies (Abrescia et al., 2004; San Martin et al., 2002).

A total of 311 sub-tomograms (200^3^ voxels) containing individual viruses were extracted from 24 uncorrected tomographic reconstructions. The EM map of PRD1 (San Martin et al., 2002, EMDB1011) was used as a starting reference for alignment of sub-tomograms, after applying a heavy low-pass filter at 1/80 Å^−1^. All alignment iterations were carried out using a soft-edged spherical mask. An initial alignment was performed allowing rotation of psi and theta in the whole icosahedral asymmetric unit, the angle phi was allowed a 360° rotation. Angles were defined according to the convention of the TOM and AV3 software packages used, as described in Forster et al. (2005). In each iteration the overall angular range searched was decreased, down to a sampling of 1°. An appropriately shaped missing wedge was applied to the Fourier transform of the rotated references in each alignment before cross-correlation with each particle. The aligned sub-tomograms were averaged, excluding those with poor cross-correlation with the reference (lower than 0.5 of the mean cross-correlation value).

Icosahedral symmetry was applied to the averaged map using Bsoft (Heymann, 2001) before using it as a reference for the next iteration.

**Fig. 5:**
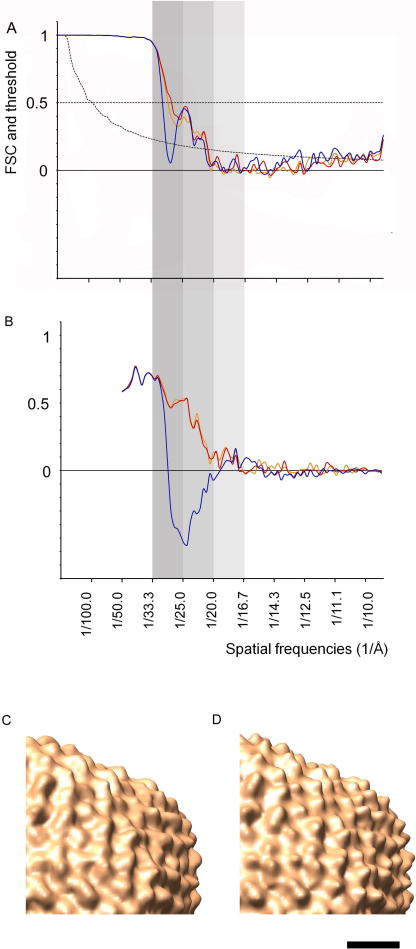
Effect of correction on averaged PRD1 sub-tomograms. (A) Fourier shell correlation between averaged maps obtained from aligned sub-tomograms split in two datasets. The 0.5 and the 3*σ* threshold curves are plotted for comparison. Blue line: uncorrected, the resolution is 29 Å at 3*σ*. Red line: corrected using the mean defocus value for all images, the resolution is 22 Å at 3*σ*. Orange line: corrected using the defocus value calculated from the relative magnification, the resolution is 22 Å at 3*σ*. (B) FSC between averaged maps obtained from aligned sub-tomograms and an appropriately scaled and aligned electron density map obtained from the available atomic model (PDB 1W8X). The colour code corresponds to that in (A). (C) Portion of the 3D map obtained from uncorrected sub-tomogram averaging. (D) Corresponding portion of the 3D map obtained from averaging of sub-tomograms after correction based on the mean defocus value. Scale bar is 100 Å.

Using this approach, a map of PRD1 was obtained from uncorrected images with a resolution of 30 Å at the 3*σ* threshold and of 31 Å at the 0.5 threshold of the FSC (Fig. 5A, blue line).

#### 3.4.2 Correction of PRD1 tomograms using the series mean defocus value

The mean defocus value was measured from the summed power spectra of each tomogram, and a range between 3 and 5.6 μm was obtained. The average mean defocus was 4 μm, this generates a CTF with its first node falling at 1/28 Å^−1^, corresponding to the local minimum observed in the FSC.

In a first correction experiment, each image in the tilt series was CTF corrected setting the defocus at the tilt axis to be the mean defocus of the series. Images were corrected using the tile-by-tile approach described above. The corrected tiles extended over 128^2^ pixels (at a tilt angle of 60° the defocus variation across a tile of this size is 0.05 μm), while FFTs were computed over 256^2^ pixels. The tilt axis direction and the tilt angle were calculated during the alignments performed with IMOD.

Sub-tomograms containing PRD1 viruses were extracted from the corrected 3D reconstructions, and an averaged map was created using parameters from an intermediate alignment step of the uncorrected data. Subsequently, further alignment iterations were carried out exactly as for the uncorrected dataset. The resolution assessed from the FSC at the 3*σ* threshold improved upon correction from 29 to 22 Å and from 30 to 27 with the 0.5 threshold criterion (Fig. 5A, red line). As expected, the dip in the FSC at 1/28 Å^−1^ corresponding to the first node of the CTF of the mean defocus is filled in after correction.

We also plotted the FSC between the averaged tomographic maps and the electron density map obtained from the atomic structure of PRD1 (Abrescia et al., 2004). We obtained a dramatic improvement upon CTF correction, with the recovery of a region of negative correlation in the FSC (Fig. 5B). An improvement can also be visually appreciated by comparing the two maps (Fig. 5C and D).

#### 3.4.3 Correcting PRD1 tomograms using the relative magnification to predict the defocus

In another experiment all 24 PRD1 tomograms were corrected using a defocus value for each image derived from its magnification relative to the other images within the same tomogram. The magnification was measured on the position of about 20 gold beads, adjusted for the tilt. The centre of mass of the gold beads used for determining the magnification must correspond to the centre of the tomogram for the defocus assessment to be correct. In our data, the average distance of the centre of mass of gold beads with respect to the centre of the tomogram was 88 pixel, leading to an error in defocus determination of 0.072 μm for an image tilted to 60°, which is not significant.

Fig. 5A, orange line, shows the FSC curves between half datasets for the viruses corrected after calculation of the defocus value from the relative magnification. The resolution does not significantly differ from the resolution of the reconstruction corrected using the mean defocus value. This indicates that the error in the determination of the defocus value for the cryo-tomographic datasets based on the relative magnification of the images is comparable to the error present when using the mean defocus value.

## 4 Discussion

In order to improve the resolution of a 3D structure obtained in single particle projects, CTF correction is routinely performed on images of cryo-specimens. The defocus value which determines the shape of the correction curve is obtained experimentally upon detection of the signal oscillations in the images power spectrum. The difficulty of detecting the signal oscillations in low SNR images has so far discouraged the implementation of CTF correction in CET. In this study we showed with simulated data that a certain margin of error in measurement of the defocus still allows the CTF to be corrected to give improvement in resolution. Strikingly, we found that an error in the defocus determination of 1.2 μm in a tilt series with mean defocus value of 4 μm allows the signal to be restored with satisfactory intensity for resolutions up to 24 Å, and an error in the order of 0.5 μm leads to a restoration of the signal up to resolutions of 17 Å.

Two methods for assigning an approximate defocus value were analysed in this study: the first corrects all images in a tilt series by using the mean defocus of all images, which can be detected in their mean power spectrum. This approach is equivalent to making the assumption that there are no *z*-shifts at the tilt axis between subsequent images. The second approach is based on the fact that a variation in defocus between images in a tilt series is reflected in a change in magnification. Applied on data of carbon film, this approach gives estimates of defocus with an average error of less than 0.5 μm.

The two methods for assigning the defocus were tested by generating 3D maps of the bacteriophage PRD1 by sub-tomogram alignment and averaging. The CTF correction of the tilted images was carried out using a tile-by-tile approach.

Datasets corrected using either method showed an improvement in resolution with respect to the uncorrected data, from 29 to 22 Å. This improvement is significant, especially in the context of fitting atomic structures into electron density maps. There was no significant difference in the performance of the two methods. This probably indicates that the estimate of defocus based on relative magnification is not as precise for cryo-images as it is in the case of images of carbon film. This may reflect larger errors in magnification measurements due to small movement of the gold beads within the cryo-sample upon exposure to the electron beam. CTF correction based on the mean defocus value over the whole tilt series leads to better results if the range of defoci is small (Supplementary Fig. S3). This implies that addition of a selection step in which only the lowest change in defocus (i.e. with lowest change in magnifications) tomograms are added to the final averaged reconstruction might be key to further resolution improvements.

The ability to align close-to-focus data is highly sample-dependent, as the contrast obtained at low defocus may not permit the alignment of sub-tomograms of smaller proteins or protein complexes. Furthermore, CTF correction on tomograms with smaller defocus values is more difficult, as the first CTF node occurs at higher frequency, where the signal intensity is lower, making it harder to detect the signal from the mean power spectrum of the tilt series. As illustrated in Supplementary Fig. S2, reconstructions from corrected tomograms collected at higher defocus values may provide better resolution than those obtained from non-corrected closer-to-focus tomograms. CTF correction of further-from-focus data may therefore present advantages for higher resolution reconstruction, when compared to using close-to-focus data.

In conclusion, clear improvements in reconstruction quality can be obtained by applying CTF correction in sub-tomogram averaging from CET. Imprecise defocus measurements are sufficient to allow useful correction, and it is possible to determine defocus from cryo-tomograms within appropriate bounds of error. This provides a framework for generating higher resolution reconstructions of protein complexes *in situ*.
