# Supplementary material for: Contrast transfer function correction applied to cryo-electron tomography and sub-tomogram averaging
Source: J Struct Biol. 2009 Nov;168(2-24):305–12. doi: 10.1016/j.jsb.2009.08.002 (PMC2806944; doi:10.1016/j.jsb.2009.08.002)
Supplement: Supplementary material — Supplementary information. [file mmc1.doc]

***Supplementary Information***

**Contrast transfer function correction applied to cryo-electron tomography and sub-tomogram averaging.**

*Giulia Zanetti^1,2^, James D. Riches^1^, Stephen D. Fuller^2^, John A. G. Briggs^1*^*

1. Structural and Computational Biology Unit, European Molecular Biology Laboratory, Meyerhofstrasse 1, Heidelberg, Germany.

2. University of Oxford, Division of Structural Biology, Wellcome Trust Centre for Human Genetics, Henry Wellcome Building for Genomic Medicine, Oxford, United Kingdom.

**Envelope function in the contrast transfer function**

The envelope function used in equation 1.0 of the introduction is reported below:


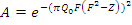


Where F is the generalised frequency
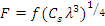
, Z the generalised defocus
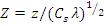
, Q_0_ the generalised half width of the illumination aperture, 0.0143 for an angular aperture of 0.1 mrad (Wade, 1978).

**Relationship between defocus and magnification**

Here, a method is described to determine the difference in the average defocus of each image of a tilt series based on the relationship between defocus and magnification. A change in the position of the specimen with respect to the objective lens results in images with different defocus values as well as different magnifications (figures S1A and B).

The defocus can be defined as the distance between the position of the object and the object plane of the objective lens (i.e. the position of the object for which the image is at focus). The difference in defocus between two images is the difference in the correspondent object positions (Δz = o_1_ - o_2_). In the common case that the condenser 2 lens is over-focussed (the beam cross-over is before the specimen, c_x_ > o_1_ and c_x_ > o_2_), it can be assumed that:

$$\left\{\begin{matrix} \frac{h}{c_{x}-o_{1}}=\tan(\alpha_{1}) \\ \frac{h}{c_{x}-o_{2}}=\tan(\alpha_{2}) \\ \frac{h_{1}^{'}}{c_{x}}=\tan(\alpha_{1}) \\ \frac{h_{2}^{'}}{c_{x}}=\tan(\alpha_{2}) \end{matrix}\right.$$

It can be derived that:

$$\frac{h_{1}}{h_{2}}=\frac{c_{x}-o_{1}+\Delta z}{c_{x}-o_{1}}$$

This conclusion is analogous to that reported by Van Duinen et al. for the case in which the defocus change is caused by a variation in the strength of the objective lens (van Duinen et al., 2005).

In a system with fixed c_x_ (unvaried Condenser 2 lens settings), defocus and magnification are linearly related. The slope of the correlation line depends mainly on the convergence of the beam on the specimen, i.e. the strength of the Condenser 2 lens. The absolute value of the gradient is higher for lower dose conditions, consistent with a higher value of c_x_ (cross-over point more distant from the specimen) in the equations above.

Magnification and defocus were measured on images of carbon film collected at condenser lens settings used in cryo-tomogram acquisition. The data (shown in figure 4 of the main text) was fitted with linear equations (y = *a*x + *b*), where the dependent variable y is the change in defocus, and x is the relative magnification between two images. In 5 datasets, we obtained the values for *a* and *b* reported below. The average value of *a* calculated over 5 experiments is -440 µm.

| *a* | *b* |
| --- | --- |
| -432 | -0.4 |
| -422 | -0.8 |
| -461 | -1.0 |
| -486 | -0.4 |
| -441 | -0.2 |


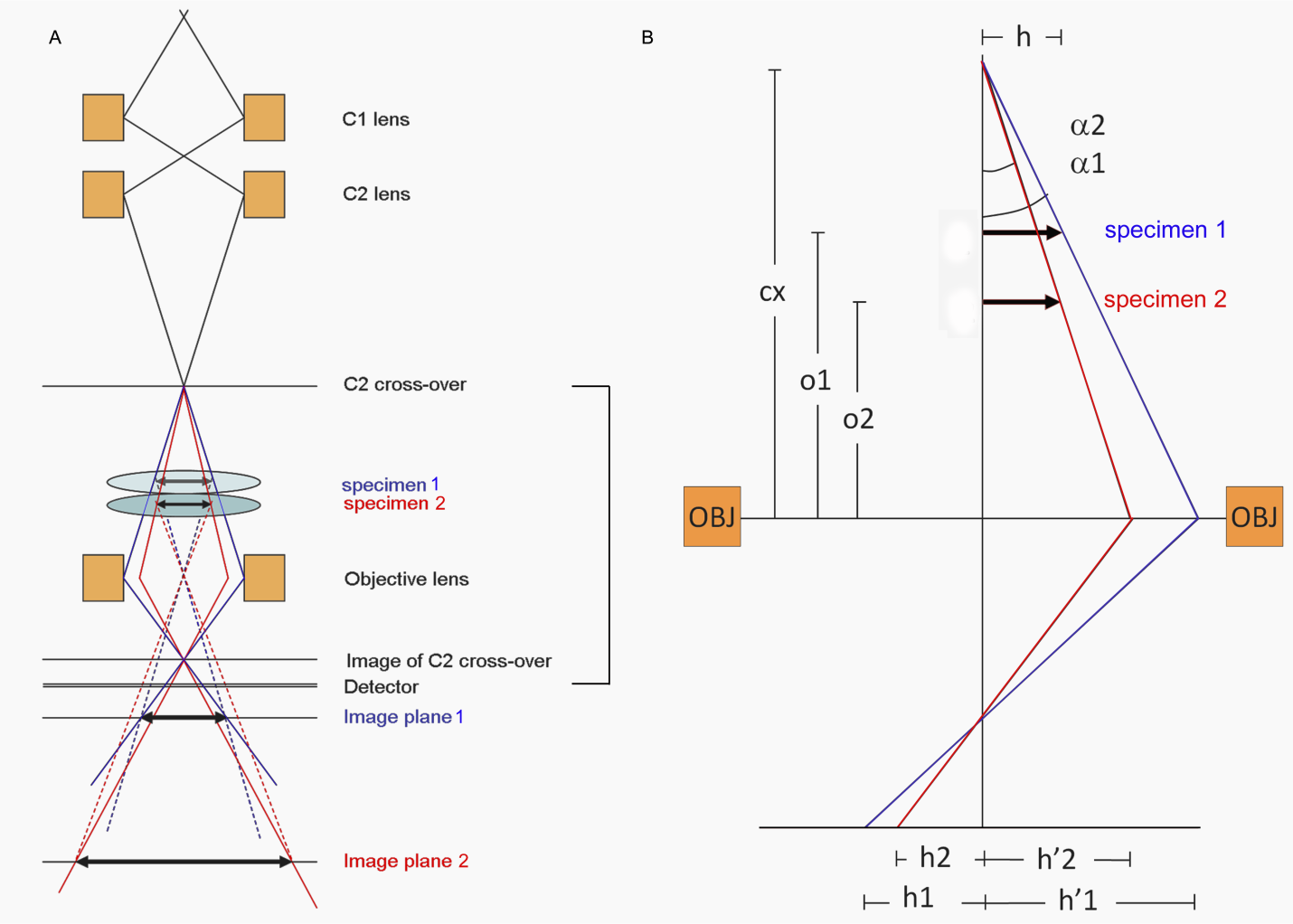


**Figure S1. Image plane shift upon movement of the specimen.**

*A) The divergent electron beam hits the specimen. The rays scattered by the specimen converge to form the image at the image plane. At the detector level the image is characterized by certain defocus and magnification. The planes identified by blue lettering correspond to the specimen in the original position. The specimen moved to a different position is represented in red. B) Zoom in the area between the Condenser 2 cross-over and the detector. For specimen 1, o_1_ represents the distance from the objective lens, h_1_/h its magnification, h’_1_ is the distance between the point where the ray hits the lens and the centre of the lens. An analogous convention is applied to specimen 2. C_x_ is the distance between the condenser 2 lens cross-over and the objective lens.*


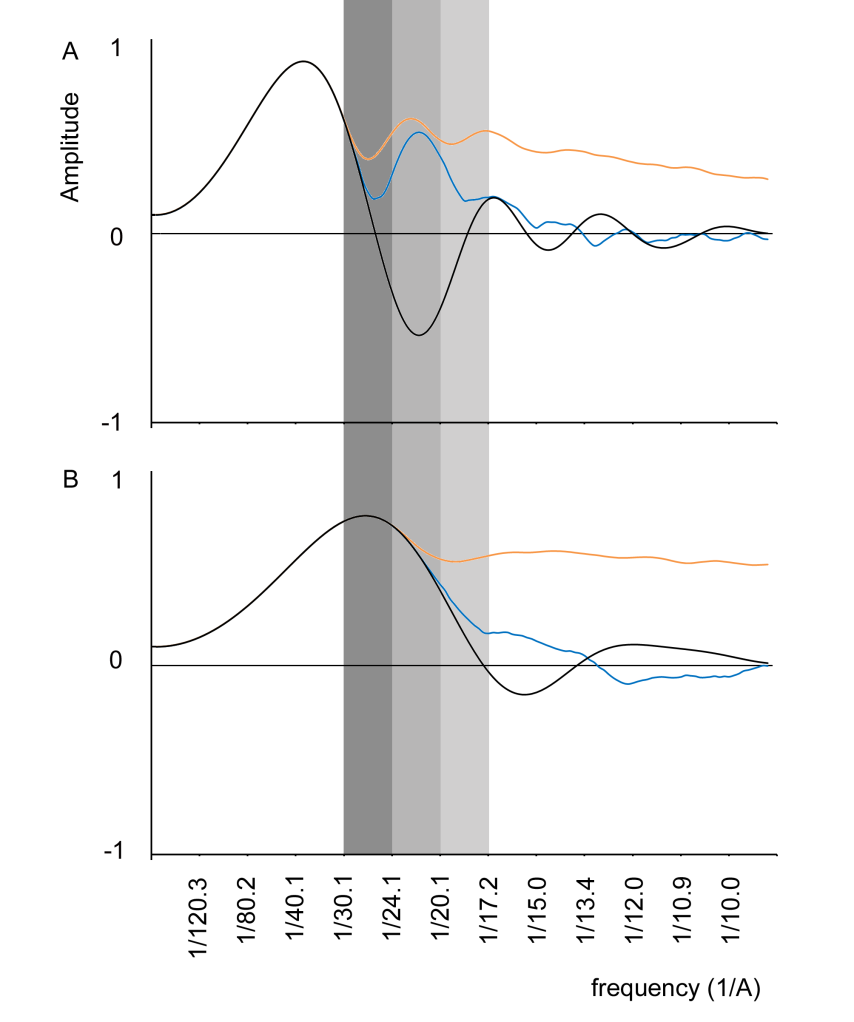


**Figure S2. Simulation of CTF in close-to-focus tomograms**

*A) The CTF of a reconstruction from 5 uncorrected tomograms with mean defocus value of 3.4 µm (black line), from 5 tomograms corrected accurately (orange line), or corrected based on the mean defocus of the series with the tilt taken into account (blue line). This panel corresponds to panel D of figure 2 and is shown here for comparison. B) The same as in A, but the mean defocus value for the 5 tomograms is 2.4 µm. Approximate correction of further-from-focus data (panel A, blue line) may yield higher resolution than using non-corrected close-to-focus data (panel B black line).*


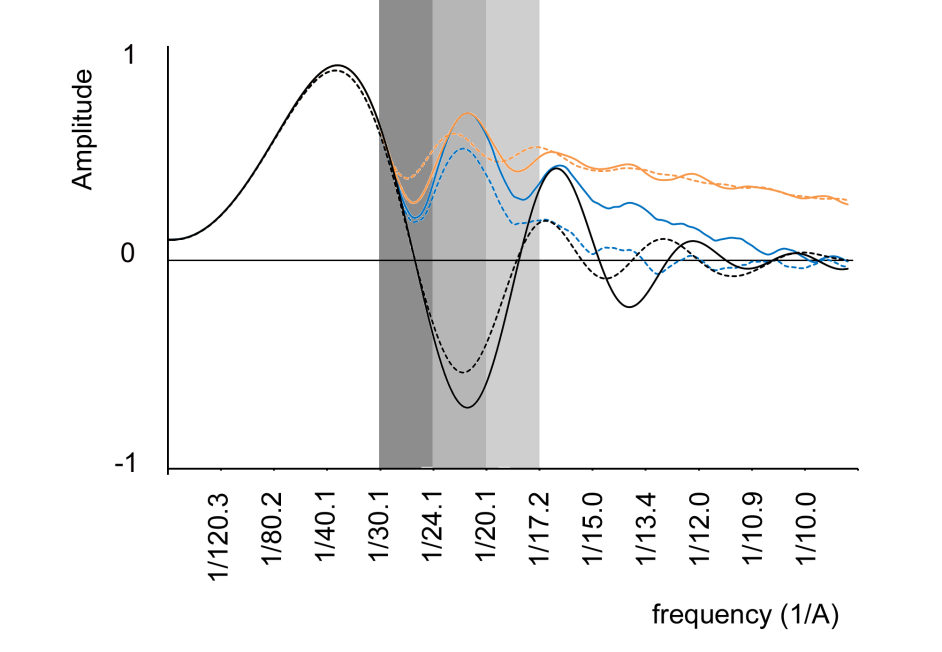


**Figure S3. Simulations of CTF for small defocus variations.**

*The CTF of a reconstruction from 5 uncorrected tomograms with mean defocus value of 3.4 µm (black lines), from 5 tomograms corrected accurately (orange lines), or corrected based on the mean defocus of the series with the tilt taken into account (blue lines). The dotted lines correspond to the solid lines in figure 2D, and represent 5 tomograms in which the maximum variation in defocus at the tilt axis is about 2.5 µm. The solid lines represent 5 tomograms in which the variation of defocus at the tilt axis is halved. Smaller variations in defocus are advantageous when the mean defocus of the series is used for correction.*

*Supplementary reference:*

van Duinen, G., M. van Heel, and A. Patwardhan, 2005. Magnification variations due to illumination curvature and object defocus in transmission electron microscopy. Optics Express 13: 9085-9093.
